# State of Medicine in America

**Published:** 1837-04

**Authors:** 


### STATE OF MEDICINE IN AMERICA.

We have received the following document since the publication of our American Report in last Number; and, as it forms an important addition to that paper, we give it here at full length.

*Table of Charges for Professional Services, revised and adopted by the Philadelphia College of Physicians, in November, 1836.*

| | Dollars. |
|---|---|
| For a single visit in a case, from | 1 to 10 |
| When detained, for each hour | 2 ... 5 |
| For an ordinary visit | 1 ... 2 |
| When to more than one person in a family, fifty cents. for each additional patient. | |
| For a visit at a time appointed by the patient or his friends | 0 ... 2 |
| For verbal advice at the physician's house | 1 ... 10 |
| For written advice | 5 ... 20 |
| For rising at night, without leaving his house | 1 ... 5 |
| For rising at night, and a visit | 5 ... 10 |
| For a first visit in consultation | 0 ... 5 |
| For subsequent visits in the same case | 1 ... 3 |
| For rising at night, and a visit in consultation | 5 ... 15 |
| In visits to distant patients, one dollar for every mile beyond the limits of the city, in addition to the ordinary charges. | |
| An extra charge may be discretionally made for travelling at night, or on account of the badness of the roads, or the inclemency of the weather. | |
| For vaccination | 0 ... 5 |
| For an ordinary case of midwifery | 10 ... 30 |
| For the application of forceps, or for turning, when, in a consultation, either shall be deemed necessary, an addition of | 0 ... 10 |
| For any indisposition in the mother or child, after the tenth day of confinement, the charge for attendance as in ordinary cases requiring medical treatment. | |
| For reducing fractures | 5 ... 10 |
| For reducing luxations | 5 ... 30 |
| For passing the catheter | 1 ... 10 |
| For removal of stone from the bladder | 100 ...200 |
| For amputation of a leg or arm | 25 ...100 |
| For amputation of a finger or toe | 5 ... 20 |
| For extirpation of large tumours | 50 ...100 |
| For extirpation of other tumours | 5 ... 30 |
| For trepanning | 25 ...100 |
| For the operation for cataract | 50 ...100 |
| ... for aneurisms,—subclavian, carotid, femoral, | 100 ...200 |
| ... for hernia | 25 ...100 |
| ... for fistula lachrymalis | 15 ... 30 |
| ... for hare-lip | 20 ... 50 |
| ... for fistula in ano | 20 ... 40 |
| ... for hydrocele | 5 ... 20 |
| ... for ascites | 10 ... 20 |

In all surgical cases, the charge for subsequent attendance to be according to the time occupied and the trouble incurred.

N.B. To enable practitioners to exhibit uniformity in the rate of charging, it is proposed that no entry shall ever be made in their account books of lower fees than those contained in the above table. If in any case, however, the physician should have reason to believe that his patient cannot pay the full amount without serious inconvenience, a deduction may be made at the end of the year, at the moment of rendering his bill, or at any other time. But the Fee-bill, as at present established by the College, being founded on a just consideration of the important services which its members are called on to perform, it is their duty to conform to it in their charges, whenever the circumstances of their patients are not such as clearly to forbid it.

